# Dataset of B-vitamins and selected one-carbon and sulfur-containing metabolites in maternal venous and paired umbilical venous and arterial plasma at elective caesarean delivery

**DOI:** 10.1016/j.dib.2026.113194

**Published:** 2026-08-27

**Authors:** Viktor Kožich, Jitka Sokolová, Antonín Pařízek, Pavlína Jirásková, Kristýna Nelicová, Jakub Krijt, Samuel Stanovský, Tomáš Honzík

**Affiliations:** a Department of Paediatrics and Inherited Metabolic Disorders, Charles University-First Faculty of Medicine, and General University Hospital in Prague, Ke Karlovu 2, 128 08 Praha 2, Czechia; b Department of Gynaecology, Obstetrics and Neonatology, Charles University-First Faculty of Medicine, and General University Hospital in Prague, Apolinářská 18, 128 08 Praha 2, Czechia

**Keywords:** Vitamin B12, Folate, Methylmalonic acid, Choline, Sulfur amino acids, Thiosulfate

## Abstract

This article describes a dataset of B-vitamins and selected one-carbon and sulfur-containing metabolites measured in maternal venous plasma and paired umbilical venous and umbilical arterial plasma collected at delivery from pregnancies completed by elective caesarean section. Maternal venous blood was collected into lithium-heparin tubes during preoperative preparation before peripheral cannula insertion, immediately placed in an ice/water bath, and processed within 60 min. Umbilical venous and umbilical arterial blood were collected at delivery and handled using the same pre-analytical protocol. Plasma samples were stored frozen at −80°C until analysis.

The dataset is organized as a sample-level spreadsheet and contains 394 sample rows from 130 Family ID values, including 130 maternal venous (MV), 132 umbilical venous (UV), and 132 umbilical arterial (UA) samples. Recorded variables include sample identifiers, maternal age, haemolysis index, icteric index, and plasma concentrations of methionine, total homocysteine, total cysteine, lanthionine, cystathionine, hypotaurine, taurine, S-sulfocysteine, thiosulfate, total γ-glutamylcysteine, total cysteinylglycine, total glutathione, total vitamin B12, active vitamin B12, methylmalonic acid, folate, glycine, serine, choline, betaine, dimethylglycine, and sarcosine.

The spreadsheet structure supports paired maternal–foetal comparisons and foetal venous–arterial comparisons. It also preserves coding for incomplete and censored observations, and threshold-coded values above the reporting range. The dataset is deposited in Mendeley Data under DOI 10.17632/8pp94xm46j.2, with the current public record. These data may be reused in perinatal metabolism research, sulfur metabolism, one-carbon metabolism, vitamin status studies, and secondary analyses of pre-analytical sample quality.

Specifications Table

**Table:** 

Subject	Health Sciences, Medical Sciences & Pharmacology
Specific subject area	Perinatal metabolism of B-vitamins, one-carbon metabolites, sulfur-containing metabolites, and maternal–foetal transfer at delivery.
Type of data	Table, Figures, Analysed data, Processed data
Data collection	Maternal venous, umbilical venous, and umbilical arterial blood were obtained in mother-foetus pairs at elective caesarean delivery between November 20, 2023 and September 24, 2024. Blood was collected into lithium-heparin tubes, placed immediately in an ice/water bath, centrifuged within 60 min, and plasma was frozen at −80°C. Analytes were measured by in-house HPLC and LC–MS/MS assays or by LC–MS/MS assays using commercial kits; vitamin B12, holotranscobalamin, and folate were determined by commercial immunoassays.
Data source location	Data were generated at the Department of Paediatrics and Inherited Metabolic Disorders (50.0698336N, 14.4286225E), the Department of Gynaecology, Obstetrics and Neonatology, and the Institute of Medical Biochemistry and Laboratory Diagnostics; all departments are organisational units of the First Faculty of Medicine, Charles University and General University Hospital in Prague, Prague, Czech Republic.
Data accessibility	Repository: Mendeley Data. Dataset citation: Kozich, Viktor (2026), “B-vitamins and selected one-carbon and sulfur-containing metabolites in maternal and cord blood”, Mendeley Data, version 2, doi:10.17632/8pp94xm46j.2. Direct URL to data: https://data.mendeley.com/datasets/8pp94xm46j/2 Instructions for accessing these data: The dataset record is publicly visible.
Related research article	None.

## 1 Value of the Data

• Only a few perinatal datasets combining matched maternal venous, umbilical venous, and umbilical arterial plasma samples collected at delivery with a broad panel of one-carbon, B-vitamin-related, choline/betaine-related, sulfur-containing, and taurine-related metabolites are publicly available. The harmonised sampling and pre-analytical handling allow direct comparison across the maternal–foetal and foetal venous–arterial compartments.
• The dataset may benefit perinatal biochemists, obstetric and neonatal researchers, researchers studying sulfur amino acid and hydrogen sulfide metabolism, investigators modelling placental transport, and groups developing reference intervals or validating biomarkers in the perinatal period.
• The data can be used to calculate maternal–foetal and foetal venous–arterial concentration gradients as surrogate indicators of placental transfer, foetal uptake, release, or metabolic utilisation of individual analytes. They may also support biomarker validation, including assessment of methylmalonic acid, total homocysteine, vitamin B12-related markers, folate-related metabolites, and sulfur-containing compounds at birth.
• These data may be reused for hypothesis generation in studies of placental transport, foetal metabolism, perinatal one-carbon metabolism, sulfur biology, taurine-related pathways, and hydrogen sulfide homeostasis. They may also be pooled with other cohorts for meta-analyses or used as comparative data for future observational or interventional studies.

## 2 Background

Maternal–foetal transfer of one-carbon metabolites, B-vitamins, and sulfur-containing amino acids is important for foetal growth because these pathways support methyl-group supply, nucleotide synthesis, amino acid interconversions, and redox homeostasis during development [1,4]. Human studies have reported maternal–cord relationships for selected biomarkers such as folate, choline, betaine, dimethylglycine, homocysteine, total vitamin B12, holotranscobalamin, glutathione-related metabolites, and individual amino acids [[2], [3], [4], [5], [6]]. However, most available studies have focused on only a few analytes or relatively small series of metabolites, and comprehensive datasets spanning one-carbon metabolites, B-vitamins, and sulfur amino acids in individually paired mother–newborn samples remain limited [[2], [3], [4], [5], [6]].

This dataset presents measurements of B-vitamins and selected one-carbon and sulfur-containing metabolites in maternal venous plasma and paired umbilical venous and arterial plasma collected at delivery. The dataset may be used for analyses of maternal–foetal concentration gradients and foetal venous–arterial differences.

## 3 Data Description

The dataset is deposited in Mendeley Data under DOI 10.17632/8pp94xm46j.2 and is listed as Version 2, published on 10 June 2026. The name of the deposited file is “Kozich maternal and cord blood.xlsx”. The workbook contains two sheets. The DATA MODEL DETAILS sheet defines individual analytes, their units and analytical methods used to generate the data. The DATA sheet contains several types of variables (see Table 1).

**Table 1:** Variable groups and data types in the DATA sheet.

**Variable group**	**Variables**	**Data type**
Pair/sample identifiers	Family ID; Sample ID	Identifier; numeric or text-coded identifier
Sample compartment	Sample type	Categorical text
Twin coding	Fetus ID	Categorical numeric code; 1 = singleton pregnancy fetus or geminus A, 2 = geminus B
Maternal data	Age of mother	Continuous numeric variable; years
Pre-analytical quality indices	Hemolysis index; Icteric index	Numeric instrument-generated quality indices
Analytes	Met; tHcy; tCys; Lth; Csth; HypT; Tau; SSC; S_2_O_3_^2−^; tGGC; tCysGly; tGSH; tB12; aB12; MMA; FOL; Gly; Ser; Chol; Bet; DMG; Sarc	Continuous quantitative concentration variables; numeric values where measured, NA for unavailable measurements and threshold-coded text for censored values where applicable

**Figure 1:**
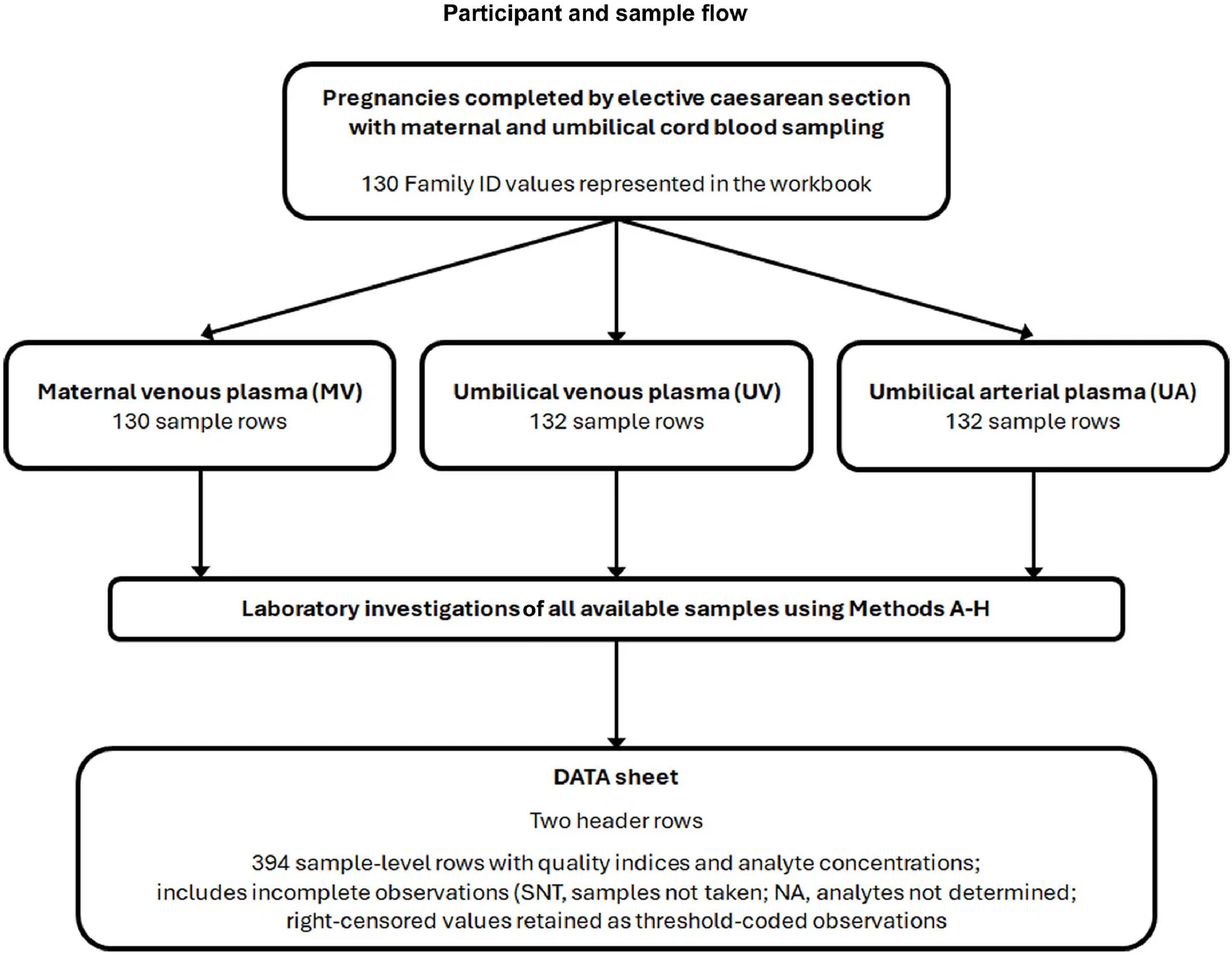
Participant and sample flow.

Identifier variables include Family ID and Sample ID. Sample type is a categorical text variable coded as MV, UV, or UA. Fetus ID is a categorical numeric code used for twin deliveries. Maternal age is a continuous numeric variable. Hemolysis and icteric indices are numeric instrument-generated quality indices. The analyte columns are continuous quantitative concentration variables reported with analyte-specific units. Missing or unavailable measurements are coded as NA (typically for low-volume samples that did not permit all analyses), sample-not-taken events as SNT (5 umbilical artery samples and 1 maternal venous sample), and values above the analytical reporting range are right-censored and retained as threshold-coded text values, such as >40 or >20 for folate, and >256 for active vitamin B12, where applicable. The DATA sheet contains two header rows: row 1 contains variable names and row 2 contains units for quantitative variables where applicable. For programmatic reuse, row 1 should be used as the column-name header and row 2 may be parsed as unit metadata or removed before analysis. The Excel workbook contains 394 sample rows in the DATA sheet after exclusion of the two header rows. The sample-level structure includes 130 maternal venous, 132 umbilical venous, and 132 umbilical arterial samples. Pregnancy-level row counts indicate 128 pregnancies represented by 3 rows and 2 pregnancies represented by 5 rows, consistent with twin coding in the Foetus ID field. Participant and sample flow is shown in Figure 1.

The dataset includes two pre-analytical quality indicators. The Haemolysis index is the automated serum index on the Cobas system (Roche Diagnostics) for haemolysis, with a critical value ≥60; plasma folate and total glutathione cannot be interpreted in samples with haemolysis index above the critical value. The Icteric index is the corresponding index for icterus, with a critical value ≥29.

The quantitative variables in the DATA sheet comprise plasma concentrations of sulfur amino acids, taurine-related metabolites, hydrogen sulfide-related metabolites, glutathione-related metabolites, vitamin B12 and folate markers, methylmalonic acid, choline-related metabolites, and selected amino acids. Most analytes are reported in µmol/L; vitamin B12 forms are reported in pmol/L, methylmalonic acid in nmol/L, and folate in µg/L.

Median concentration and ranges of analytes in maternal venous plasma and plasma samples obtained from umbilical cord vessels were calculated using Excel and are shown in Table 2. Data were calculated from a dataset Kozich maternal and cord blood.xlsx after excluding data on folate and GSH in samples with haemolysis index >60; we also excluded one family with a genetic diagnosis of ACS3 deficiency and 2 mother-foetus pairs with twin pregnancies.

**Table 2:** Median concentrations and ranges of analytes in maternal venous plasma and paired umbilical venous and arterial plasma

Metabolite	Maternal venous plasma		Umbilical venous plasma		Umbilical arterial plasma	
	N	Median (Range)	N	Median (Range)	N	Median (Range)
***Methionine cycle and transsulfuration***						
**Methionine,***µmol/L*	124	18.5 (10.8–33.1)	122	27.1 (17.9–44.0)	82	26.0 (18.3–59.0)
**Sarcosine,***µmol/L*	123	0.35 (0.03–0.97)	122	0.60 (0.06–1.6)	82	0.67 (0.38–1.1)
**Total homocysteine,***µmol/L*	125	6.3 (3.7–17.1)	124	5.7 (3.3–13.0)	102	5.7 (2.8–10.1)
**Cystathionine,***µmol/L*	124	0.157 (0.057–0.844)	122	0.295 (0.079–2.65)	82	0.318 (0.092–2.89)
**Total cysteine,***µmol/L*	125	186 (127–271)	124	177 (130–238)	102	175 (129–242)
***Markers of folate and vitamin B12 saturation***						
**Folate,***µg/L*^1^	114	15.5 (1.8– >40)	115	21.4 (6.6– >40)	49	23.5 (2.9– >40)
**Total vitamin B12,***pmol/L*	124	223 (85–509)	126	292 (74–970)	115	287 (98–1007)
**Active vitamin B12,***pmol/L*^2^	122	64 (16–218)	124	123 (48– >256)	96	109 (38– >256)
**Methylmalonic acid,***nmol/L*	125	188 (51–615)	127	319 (143–1110)	120	322 (151–1100)
***Homocysteine remethylation and one-carbon metabolism***						
**Choline,***µmol/L*	123	20 (9–51)	120	38 (20–101)	90	37 (19–95)
**Betaine,***µmol/L*	123	11 (6–20)	120	26 (15–41)	90	29 (11–46)
**Dimethylglycine,***µmol/L*	123	2.2 (1.0–8.5)	120	3.4 (2.1–11.4)	90	3.6 (2.1–12.5)
**Glycine,***µmol/L*	124	129 (75–236)	122	216 (136–398)	82	236 (135–503)
**Serine,***µmol/L*	124	78 (48–140)	122	125 (85–203)	82	135 (75–258)
***Glutathione-related metabolites***						
**Total glutathione,***µmol/L*	114	3.8 (2.2–8.0)	114	0.83 (0.32–8.8)	46	0.93 (0.41–3.4)
**Total cysteinylglycine,***µmol/L*	125	21.3 (14.2–39.9)	124	19.0 (12.7–45.1)	102	22.7 (14.5–60.0)
**Total γ-glutamylcysteine,***µmol/L*	125	3.3 (0.87–5.9)	124	1.3 (0.77–2.7)	102	1.5 (0.85–6.2)
***Taurine-related metabolites***						
**Hypotaurine,***µmol/L*	123	0.34 (0.004–1.1)	120	0.91 (0.24–3.3)	90	1.1 (0.25–4.0)
**Taurine,***µmol/L*	123	33 (13–55)	120	120 (59–548)	90	125 (32–504)
***Hydrogen sulfide synthesis and catabolism***						
**Lanthionine,***µmol/L*	124	0.061 (0.018–0.126)	122	0.097 (0.056–0.192)	82	0.097 (0.056–0.189)
**Thiosulfate,***µmol/L*	123	0.93 (0.19–7.1)	121	0.78 (0.12–1.9)	87	0.80 (0.23–2.9)
**S-sulfocysteine,***µmol/L*	121	0.47 (0.05–2.5)	109	0.13 (0.004–1.2)	86	0.23 (0.02–2.3)

Column N shows the number of samples used for the calculation.

¹ The upper limit of quantification for folate was 40 µg/L for samples diluted according to the manufacturer’s protocol that remained above the upper quantification limit, and 20 µg/L for samples with insufficient volume for repeat analysis after dilution.

² The upper limit of quantification for active vitamin B12 is 256 pmol/L.

**Figure 2:**
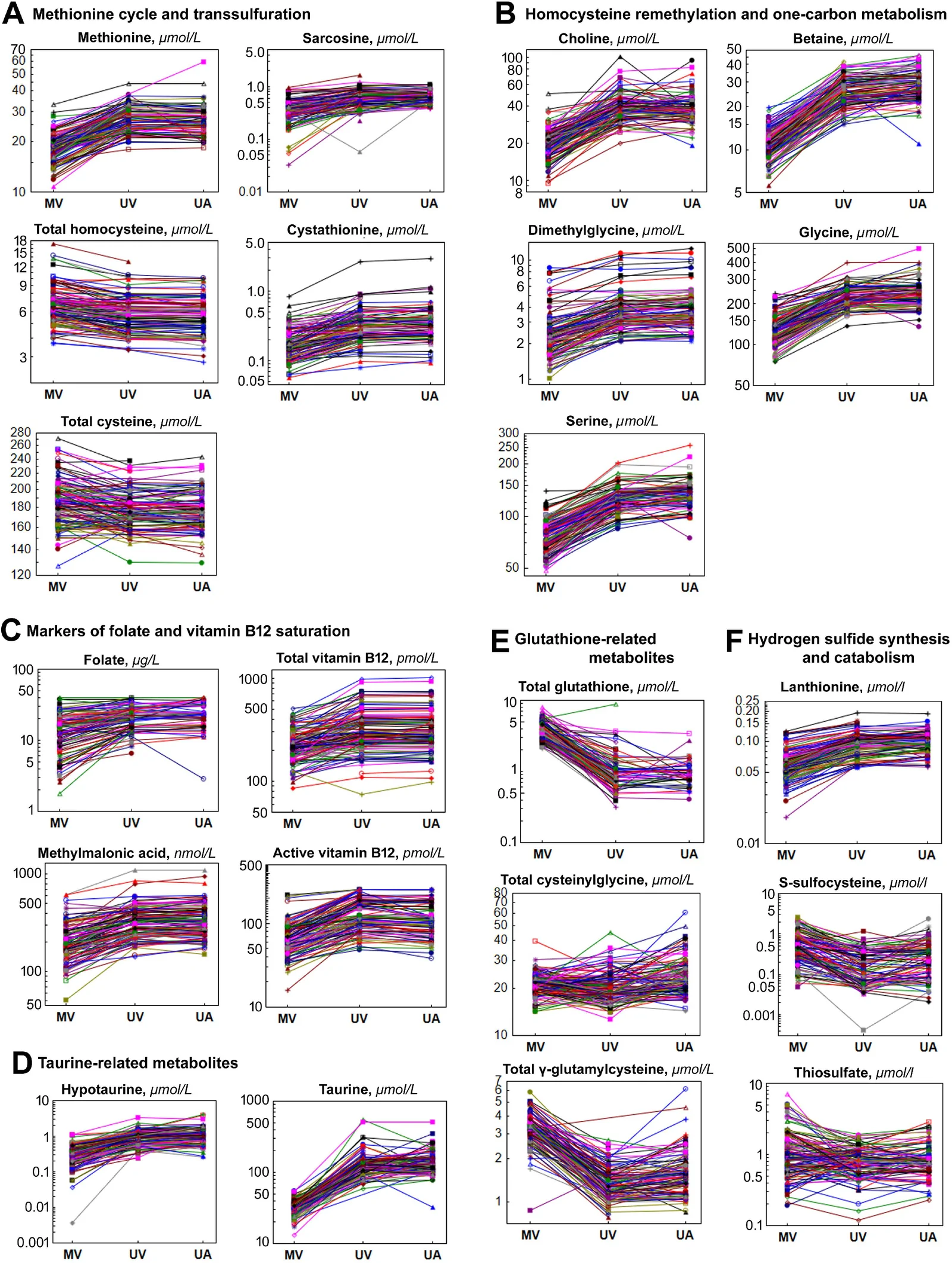
Concentration of analytes in maternal and umbilical cord blood. Individual data after the exclusions described in the footnote to Table 2. MV, maternal venous plasma, UV, umbilical venous plasma; UA, umbilical arterial plasma.

Individual analyte profiles across maternal venous, umbilical venous, and umbilical arterial plasma (see Figure 2) illustrate differences between the maternal and foetal circulations. The figure was generated using the dataset described above, after excluding folate and GSH data from samples with haemolysis index >60, one family with a genetic diagnosis of ACS3 deficiency, and two mother–foetus pairs from twin pregnancies. Data were visualized using TIBCO Statistica version 14.

## 4 Experimental Design, Materials and Methods

The dataset was generated at the First Faculty of Medicine, Charles University and General University Hospital in Prague, Czech Republic. Study subjects were recruited between November 20, 2023, and September 24, 2024. The cohort comprised 130 mother–infant pairs from pregnancies completed by elective caesarean section.

Inclusion criteria for the study were: (a) gestational age ≥37 weeks and (b) elective caesarean section for cephalopelvic disproportion, previous caesarean section(s), abnormal foetal presentation, non-progressing labour without signs of foetal hypoxia, or maternal request. Exclusion criteria included maternal pregnancy-related or internal comorbidities during pregnancy, such as autoimmune disease, endocrine disease, type 1 or type 2 diabetes mellitus, gestational diabetes requiring treatment other than diet, cardiovascular disease, hypertension, preeclampsia, acute infectious disease at the time of delivery, chronic infectious disease, renal disease, gastrointestinal or liver disease, and malignancy. Foetal exclusion criteria included foetal growth restriction, foetal overgrowth, abnormal cardiotocography before delivery, prenatally diagnosed congenital malformations or chromosomal abnormalities, placentation disorders, and prelabour rupture of membranes. Data on vitamin intake and dietary patterns prior to the start of labour were not available.

During preoperative preparation, 3 mL of maternal venous blood was collected into lithium-heparin tubes using a closed blood collection system prior to peripheral venous cannulation. Samples were placed immediately in an ice-water bath. Within 60 minutes of collection, plasma was separated by centrifugation at 2,000 × g for 5 min at room temperature, and the entire volume of separated plasma was frozen at −80°C until further analyses. The umbilical cord was clamped 60 seconds after delivery. Umbilical venous and arterial blood samples (1-2 mL) were collected by needle puncture into lithium-heparin tubes within 1-2 minutes after clamping. Umbilical blood samples were processed by the same protocol as maternal blood samples.

Samples were analysed in batches by the methods described below. Maternal and foetal samples from one family were processed in the same batch. Due to the limited sample volume, only single measurements were carried out. Operators were blinded to sample identity. Quality-control procedures followed the standard operating procedures of the respective analytical platforms and included calibration and internal quality-control materials according to the manufacturers’ instructions or in-house laboratory validation procedures, as applicable.

The DATA MODEL DETAILS sheet defines eight analytical methods. Analytical performance was determined experimentally for in-house methods and taken from kit inserts for commercial kits (for details see Table 3). Method A measured total aminothiols by HPLC with fluorescent detection using tris(2-carboxyethyl)phosphine and 7-fluorobenzofurazan-4-sulfonic acid ammonium salt [7]. Method B measured amino acids and thioethers by LC–MS/MS using the MetAmino Kit (Chromservis s.r.o., Czech Republic). As published before, method C measured betaine/choline- and taurine-related analytes by in-house LC–MS/MS and method D measured inorganic sulfur compounds by in-house HPLC with monobromobimane derivatization [8]. Method E measured methylmalonic acid with the ClinMass LC-MS/MS Complete Kit (RECIPE, Germany). Method F measured total vitamin B12 by electrochemiluminescence immunoassay using Elecsys Vitamin B12 II on COBAS 8000 module e801 (Roche Diagnostics). Method G measured holotranscobalamin by chemiluminescence immunoassay using Active-B12 on Alinity i (Abbott). Method H measured folate by electrochemiluminescence immunoassay using Elecsys Folate III on COBAS 8000 module e801 (Roche Diagnostics).

**Table 3:** Performance of analytical methods

		LOD	LLOQ	Within-run CV	Between-run CV
				%	%
**Method A**	**Total aminothiols**	µmol/L			
tHcy	Total homocysteine	0.075	0.25	1.9	3.8
tCys	Total cysteine	0.3	1	2.3	3.9
tGGC	Total gamma-glutamylcysteine	0.015	0.05	2.5	8.4
tCysGly	Total cysteinylglycine	0.06	0.2	1.2	3.2
tGSH	Total glutathione	0.05	0.17	1.9	13
**Method B**	**Amino acids and thioethers**	µmol/L			
Met	Methionine	0.003	0.01	N.D.	9.4
Lth	Lanthionine	0.001	0.003	N.D.	7
Csth	Cystathionine	0.001	0.003	N.D.	6.4
Gly	Glycine	0.002	0.005	N.D.	6.5
Ser	Serine	0.002	0.008	N.D.	2.8
Sarc	Sarcosine	0.004	0.012	N.D.	9.9
**Method C**	**Methylglycines and taurine**	µmol/L			
HypT	Hypotaurine	0.27	0.9	6.3	10.8
Tau	Taurine	0.7	2.3	3.3	4.2
SSC	S-sulfocysteine	0.038	0.125	7.2	8
Chol	Choline	0.021	0.071	2.4	5
Bet	Betaine	0.035	0.116	2.2	5.7
DMG	Dimethylglycine	0.094	0.309	2.5	5
**Method D**	**Inorganic sulfur compounds**	µmol/L			
S_2_O_3_^2−^	Thiosulfate	0.05	0.15	3.6	N.D.
**Method E**	**MMA**	nmol/L			
MMA	Methylmalonic acid	15	25	3.6	6
**Method F**	**Total B12**	pmol/L			
	Total vitamin B12	74	111	<3	<5
**Method G**	**Active B12**	pmol/L			
aB12	Active vitamin B12 (holotranscobalamin)	2	4	<4	<6
**Method H**	**Folate**	µg/L			
FOL	Folate	1.2	2	<7	<10

Data for in-house methods were obtained previously in experiments unrelated to this publication; data for methods using commercial kits were obtained from the manufacturers’ kit inserts.

## Limitations

The dataset represents a single obstetric setting, namely elective caesarean delivery for maternal indications, and does not cover all delivery modes or pregnancy phenotypes. The spreadsheet includes incomplete observations, sample-not-taken entries, and threshold-coded values above the assay reporting range. Interpretation of some analytes may also be constrained by sample quality, as haemolysis and icteric indices vary across samples and critical thresholds are defined in the data model.

## Ethics Statement

The study was approved by the Ethics Committee of the General University Hospital in Prague (approval No. EK VFN 31/21). All participating women provided written informed consent. The study was conducted in accordance with the Declaration of Helsinki.

## Credit Author Statement

**Viktor Kožich:** Conceptualization, Methodology, Supervision, Validation, Funding acquisition, Writing – original draft. **Jitka Sokolová:** Methodology, Investigation, Data curation, Validation, Formal analysis, Visualization, Writing – review & editing. **Antonín Pařízek:** Conceptualization, Investigation, Validation, Writing – review & editing. **Pavlína Jirásková:** Investigation, Resources, Data curation, Writing – review & editing. **Kristýna Nelicová:** Methodology, Investigation, Data curation, Writing – review & editing. **Jakub Krijt:** Methodology, Investigation, Writing – review & editing. **Samuel Stanovský:** Conceptualization, Methodology, Writing – review & editing. **Tomáš Honzík:** Conceptualization, Funding acquisition, Supervision, Writing – review & editing.

## Declaration of Generative AI and AI-assisted Technologies in the Writing Process

During the preparation of this work and its revision, the authors used Microsoft Copilot to improve language, readability, text organization, and consistency of the manuscript and to assist with alignment to the journal template. After using this tool, the authors reviewed and edited the content as needed and take full responsibility for the content of the publication.

## Data Availability

Mendeley DataB-vitamins and selected one-carbon and sulfur-containing metabolites in maternal and cord blood (Original data).
